# Genome-wide transcriptome analysis shows extensive alternative RNA splicing in the zoonotic parasite *Schistosoma japonicum*

**DOI:** 10.1186/1471-2164-15-715

**Published:** 2014-08-26

**Authors:** Xianyu Piao, Nan Hou, Pengfei Cai, Shuai Liu, Chuang Wu, Qijun Chen

**Affiliations:** MOH Key Laboratory of Systems Biology of Pathogens, Institute of Pathogen Biology, Chinese Academy of Medical Sciences & Peking Union Medical College, Beijing, People’s Republic of China; Key Laboratory of Zoonosis, Ministry of Education, Institute of Zoonosis, Jilin University, Changchun, People’s Republic of China

## Abstract

**Background:**

*Schistosoma japonicum* is a pathogen of the phylum Platyhelminthes that causes zoonotic schistosomiasis in China and Southeast Asian countries where a lack of efficient measures has hampered disease control. The development of tools for diagnosis of acute and chronic infection and for novel antiparasite reagents relies on understanding the biological mechanisms that the parasite exploits.

**Results:**

In this study, the polyadenylated transcripts from the male and female *S. japonicum* were sequenced using a high-throughput RNA-seq technique. Bioinformatic and experimental analyses focused on post-transcriptional RNA processing, which revealed extensive alternative splicing events in the adult stage of the parasite. The numbers of protein-coding sequences identified in the transcriptomes of the female and male *S. japonicum* were 15,939 and 19,501 respectively, which is more than predicted from the annotated genome sequence. Further, we identified four types of post-transcriptional processing, or alternative splicing, in both female and male worms of *S. japonicum*: exon skipping, intron retention, and alternative donor and acceptor sites. Unlike mammalian organisms, in *S. japonicum*, the alternative donor and acceptor sites were more common than the other two types of post-transcriptional processing. In total, respectively 13,438 and 16,507 alternative splicing events were predicted in the transcriptomes of female and male *S. japonicum*.

**Conclusions:**

By using RNA-seq technology, we obtained the global transcriptomes of male and female *S. japonicum*. These results further provide a comprehensive view of the global transcriptome of *S. japonicum.* The findings of a substantial level of alternative splicing events dynamically occurring in *S. japonicum* parasitization of mammalian hosts suggest complicated transcriptional and post-transcriptional regulation mechanisms employed by the parasite. These data should not only significantly improve the re-annotation of the genome sequences but also should provide new information about the biology of the parasite.

**Electronic supplementary material:**

The online version of this article (doi:10.1186/1471-2164-15-715) contains supplementary material, which is available to authorized users.

## Background

Human schistosomiasis, which is second only to malaria in terms of morbidity and mortality, is a chronic debilitating disease caused by infections of *Schistosoma* species that vary depending on the endemic region of the parasites [1]. Three principal *Schistosoma* species can infect humans and cause severe diseases: *Schistosoma japonicum*, *Schistosoma mansoni*, and *Schistosoma haematobium. S. japonicum* is the causative agent of zoonotic schistosomiasis, affecting millions of people in several East and Southeast Asian countries. Despite the availability of a highly effective chemotherapeutic drug (Praziquantel), the high re-infection rates in humans and animals plus the requirement of frequent administration of the agent still limits the overall success of chemotherapy and disease control efforts. Novel targets for drug and vaccine development remain to be defined for optimal treatment and disease prevention; however, the lack of knowledge about this parasite’s biology remains a hurdle. Schistosoma parasites can persist in a mammalian host for decades in the presence of the host immune system, and current knowledge about the mechanism of parasitization is still fragmented. What is known is that the successful host-evasion mechanisms of the parasite involve the inert tegument that covers the surface in most developmental stages, the recruitment of host components to the surface, and the expression of various antigens and immune-regulating factors [2–5].

Schistosoma parasites have a complicated developmental and biological cycle. They are among the few platyhelminth parasites to adopt a dioecious lifestyle and possess heteromorphic sex chromosomes. The genome of *S. japonicum* contains eight pairs of chromosomes comprising seven pairs of autosomes and one pair of sexual chromosomes, with an estimated 397 Mb containing primarily 13,469 protein-coding sequences [6, 7] that account for 4% of the genome. The decoding and availability of the genome sequences of the three most pathogenic parasites, *S. mansoni*, *S. japonicum*, and *S. haematobium*, has proved pivotal for the systematic dissection of the parasite biology [7–10].

**Figure 1:**
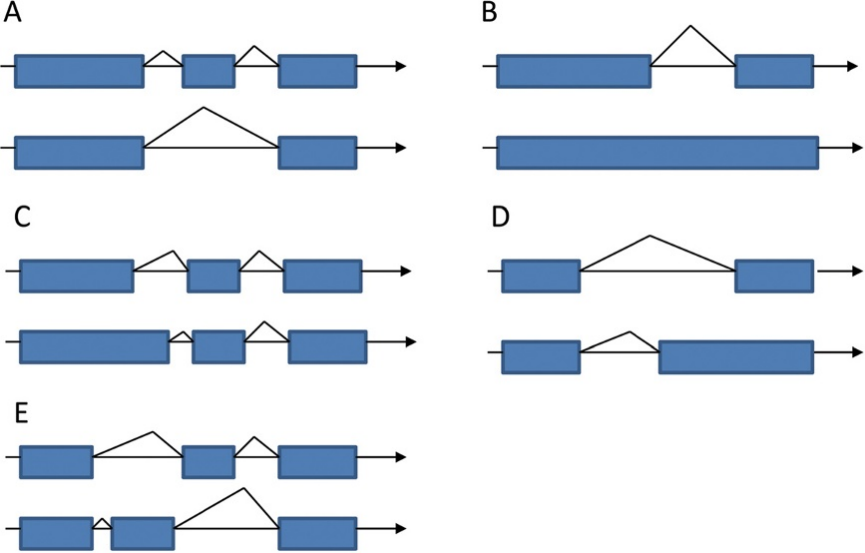
**Schematic illustration of alternative splicing. A)** Exon skipping. Gene A forms two different transcripts; the first transcript has a new exon compared to the second transcript, the new exon is an inclusive exon, and the other two exons are constitutive. **B)** Intron retention. Gene B forms two different transcripts; the second transcript is a new exon formed from retained intron and exons on both sides. **C)** Alternative donor site. Gene C forms two different transcripts; the difference is one exon of an alternative 5′ splice site of the second transcript extended. **D)** Alternative acceptor site. Gene D forms two different transcripts; the difference is one exon of the alternative 3′ splice site of the second transcript extended. **E)** Mutually exclusive exon. Gene E forms two different transcripts; the different exon is an inclusive exon, the same exon is a constitutive exon, and two transcripts have different inclusive exons.

Deep transcriptome sequencing (also called RNA-Seq) with next-generation sequencing technologies has provided unprecedented opportunities to investigate the genome-wide transcriptional property of many species [11–14]. This technique allows for the survey of the entire transcriptome in a very high-throughput and quantitative manner, making it possible to identify exons and introns, map their boundaries and the 5′ and 3′ ends of genes, and understand the complexity of genome organization and activity comprehensively. A majority of eukaryotic protein-coding genes contain intron sequences that must be removed by splicing after transcription from the DNA templates. However, some pre-mRNAs can be processed alternatively by the splicing out or retention of the transcript regions of exons or introns. This alternative splicing allows individual genes to produce two or more variant mRNA templates, which in many cases encode functionally distinct proteins. Alternative splicing is an integrated process in regulation of gene transcription and expression and results in structural and functional diversity of molecules [15]. Because of the powerful readout of RNA-seq, which can generate many sequence reads that span exon–exon junctions, RNA transcripts generated from different splicing events can be identified [12, 13, 16]. So far, five basic modes of alternative splicing are generally recognized: exon skipping (ES), mutually exclusive exons, alternative donor sites (ADS), alternative acceptor sites (AAS), and intron retention (IR) [17, 18]. ES, also called exon cassette, indicates that an exon is spliced out from the primary transcript and occurs most commonly in mammalian cells [19]. In the event of mutually exclusive exons, only one of the two exons is retained in mRNAs after splicing. An ADS results when an alternative 5′ splice junction (called the donor site) is used, leading to a change in the 3′ boundary of the upstream exon. An AAS arises when an alternative 3′ splice junction (acceptor site) is used, leading to a change in the 5′ boundary of the downstream exon (Figure 1). IR occurs when a sequence is spliced out as an intron or simply retained and is distinguished from ES because introns do not flank the retained sequence. The retained transcript of the intron region in most cases encodes amino acids in-frame with the neighboring exons [19]. Recent results have suggested that schistosomes create multiple protein variants by splicing micro-exon gene transcripts, which might be involved in immune evasion mechanisms [20]; however, the general feature of alternative splicing in the parasites remains understudied. Here, we investigated the alternative splicing of transcripts in both male and female *S. japonicum* after deep RNA sequencing. We found that the gene transcripts were diversely processed and that four types of RNA splicing were identifiable after transcription of the genome.

## Methods

### Parasites and RNA purification

*Schistosoma japonicum*–infected *Oncomelania hupensis* were purchased from Jiangxi Institute of Parasitic Disease, Nanchang, China. Cercariae were freshly shed from the infected snails. One New Zealand white female rabbit was percutaneously infected with ~1,500 *S. japonicum* cercariae, as described previously [21]. Mature adult parasites were isolated at 6 weeks post-infection from the rabbit by flushing the blood vessels with phosphate-buffered saline, as described previously [5, 22–24]. Male and female parasites were manually separated with the aid of a light microscope. Total RNA from the parasites was purified with Trizol reagent (Invitrogen, CA, USA), and contaminating genomic DNA was removed using the RNase-Free DNase Set (Qiagen, Germany). RNA quantification and quality were examined with a Nanodrop ND-1000 spectrophotometer (Nanodrop Technologies, Wilmington, DE, USA) and standard agarose gel electrophoresis. All RNA samples were stored at -80°C until use.

### Library preparation and sequencing

Polyadenylated RNA samples from adult male and female *S. japonicum* parasites were isolated from total RNA using oligo-(dT) conjugated magnetic beads (Dynabeads®, Invitrogen, CA, USA). The mRNA was interrupted into short fragments by adding the fragmentation buffer provided by the manufacturer (Illumina RNA-seq kit, part no. 1004898). With these short fragments as templates, random hexamer primers were used to synthesize the first-strand cDNA. The second-strand cDNA was synthesized using buffer, dNTPs, RNase H, and DNA polymerase I, respectively. Short fragments were purified following instructions accompanying the kit (QiaQuick PCR Purification Kit, Qiagen, Germany), and double-stranded cDNAs were end-repaired according to manufacturer-recommended protocols, followed by connection with Illumina adapters (Illumina RNA-seq kit, part no. 1004898). The fragments were first amplified by PCR. Purified cDNA fragments were pooled and indexed and loaded onto one lane of an Illumina GA IIX flow cell. A total of 75 pair-end sequencing cycles were carried out. Cluster formation, primer hybridization, and pair-end sequencing were performed according to the provided protocols [25].

### Sequence analysis

Low-quality reads (more than half of the bases had a quality value less than 5), reads in which unknown bases represented more than 10%, and adapter sequences were removed from the reads, and the clean reads were mapped onto the *S. japonicum* genome of SGST, ( http://lifecenter.sgst.cn/schistosoma/en/schdownload.do) by TopHat (version v2.0.4; default parameters were used) [26], then assembled with Cufflinks (version v2.0.2) [27] to construct unique transcript sequences using the parameter: -g –b –u –o (-g/–GTF-guide: use reference transcript annotation to guide assembly; –b/–frag-bias-correct: use bias correction-reference fasta required; –u/–multi-read-correct: use ‘rescue method’ for multi-reads; –o/–output-dir: write all output files to this directory). The Cufflinks assembler is freely available at http://cufflinks.cbcb.umd.edu/. Cuffcompare [27] was used to compare the assembled transcripts of each library to the referenced annotated genes and build a non-redundant transcript dataset among the libraries. Then, Cuffdiff was used to find significant changes in gene expression level [27]. We used FDR to correct P values and obtained Q values; for Q value ≤5%, we considered the genes to be differentially expressed (Additional file 1). Several Perl scripts were written to summarize the splicing forms in each library. The following algorithms were used to detect alternative splicing events. First, junction sites, which give information about boundaries and combinations of different exons in a transcript, were detected by TopHat (with all default parameters). Then, all junction sites of the same gene were used to distinguish the type of alternative splicing event [26] (Additional file 2: Figure S1 and Figure 1).

### Functional annotation and classification

Transcripts were first compared using the Kyoto Encyclopedia of Genes and Genomes database (KEGG, release 58) [16] with BLASTX [28] at E values ≤ 1e-10. A Perl script was used to retrieve KO (KEGG Ontology) information from the Blast result and establish pathway associations between UniGene and the database.

InterPro [29] domains were annotated by InterProScan [30] (Release 27.0), and functional assignments were mapped onto Gene Ontology (GO) [31]. WEGO [32] was employed to do GO classification and draw the GO tree. The significance analysis of functional pathways was performed using IDEG6 [33].

To identify pseudogenes in the *S. japonicum* genome, we used PseudoPipe [34]. The assembled transcripts that fell into or included the predicted position of pseudogenes were designated as pseudogenes. WEGO was used for the GO classification.

### Non-coding RNA annotation

Rfam [35] (Release 10.1) databases were used to annotate the non-coding transcripts. The assembled novel transcripts were compared to Rfam by Blast at E values ≤ 1e-10.

### Verification of alternative splicing transcripts by RT-PCR and sequencing

Genomic DNA of *S. japonicum* (adult male and female worms) was purified with the DNeasy Blood & Tissue Kit (Qiagen, Germany) according to the manufacturer’s instructions. Total RNA was prepared using TRIzol reagent (Invitrogen), as previously described [21], and contaminating genomic DNA was removed with the RNase-Free DNase Set (Ambion). PCR was conducted in triplicate, and each reaction involved 35 amplification cycles on an Applied Biosystems 9700 PCR system (Applied Biosystems, Foster City, CA, USA). The 20 μl reaction system contained 50 ng of total RNA (50 ng RNA was used for the first-strand synthesis step) or 80 ng DNA, 0.5 μM of each primer, and 10 μl of Premix Ex Taq (version 2.0, TaKaRa). The reaction conditions were as follows: 94°C for 3 min; 35 cycles of 94°C, 30 s; 55°C, 30 s; and 72°C, 90 s; and then 10 min at 72°C. An 8 μl aliquot of each PCR sample was then subjected to electrophoresis in a 1.5% agarose gel. The RT-PCR primer sequences are listed in Additional file 3: Table S1.

## Results and discussion

### Identification of a large number of novel transcripts from un-annotated genome loci following deep sequencing of the *S. japonicum*transcriptome

In this study, we determined the transcriptomes of male and female adult worms of *S japonicum* by high-throughput RNA-seq with poly-A–purified RNA samples. A total of 8,112,913 and 8,260,474 paired reads were obtained, with a total length of 1,216,936,950 and 1,239,071,100 bp from female and male worms, respectively (Table 1, Additional file 4). The number of predicted genes of female and male worms of *S. japonicum* was 15,939 and 19,501, respectively, which was more than that predicted based on the genome sequence [6, 7]. Of the 15,939 genes predicted in the female parasite, a total of 10,087 were known and 5,852 were novel, while of the 19,501 predicted genes in the male parasite, a total of 10,469 were known and 9,032 were novel. The number of predicted transcripts in the two libraries of female and male parasites was 21,009 and 25,706, respectively, with 14,301 known and 6,708 novel transcripts in females and 18,931 known and 6,775 novel transcripts in males. The finding of so many novel transcripts should assist with the upgrade or reassembling of the genome sequence of *S. japonicum* [2]. However, the novel transcripts may also be generated by alternative post-transcriptional RNA processing or alternative splicing [36]. Indeed, we found 3,905 multi-transcription loci in female and 4,677 in male parasites, with about 1.32 transcripts per locus in both sexes (Table 1). Thus, the general transcription activity of both sexes was diverse but in a similar manner, confirming earlier studies [21, 36, 37]. However, the sequence data generated from this study was much more than that of earlier studies [37]. This was likely due to the approach applied in this study which is technically advanced than the digital gene expression technique. All sequence data have been deposited in the database ( http://www.ncbi.nlm.nih.gov/geo/query/acc.cgi?) with an accession number of GSE58564.

**Figure 2:**
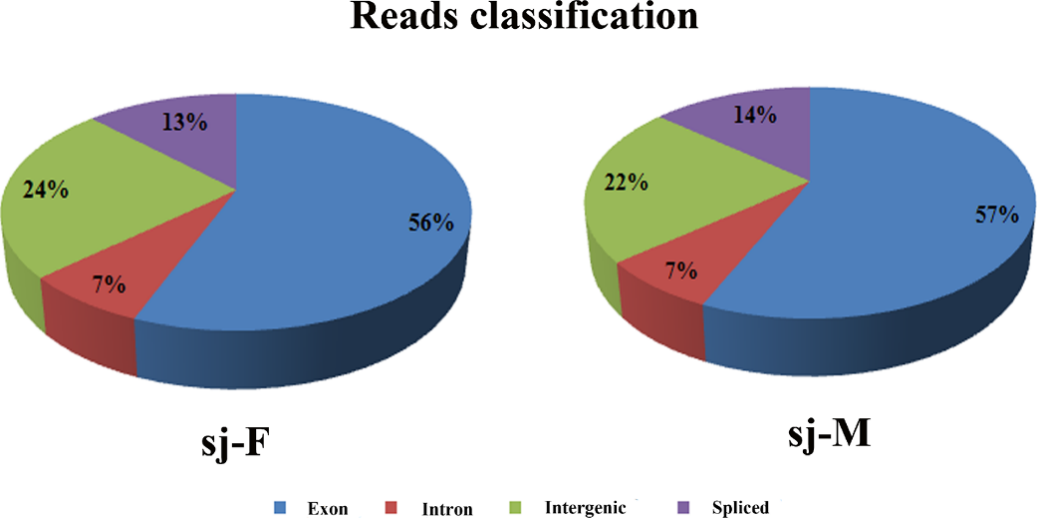
**Proportions of sequence reads (transcripts) generated from different genetic regions in the genomes of female and male** ***S. japonicum*** **.** More than 50% of the transcripts were generated from exons while transcripts from intron, intergenic regions, and splicing events were around 7%, 22%, and 13%, respectively.

The sequence reads can be classified into four types: exons, introns, intergenic, and spliced. The proportions of transcripts in female parasites from exons, introns, and intergenic loci were 56%, 7%, and 24%, respectively, and 13% of the transcripts were generated by alternative splicing (Figure 2). Similarly, in male parasites, the percentages of transcripts from exons, introns, and intergenic loci were 57%, 7%, and 22% respectively; 14% of the transcripts were generated by alternative splicing (Figure 2).

**Table 1:** **Summary data of the transcriptome analysis**

	Sj-F	Sj-M
Number of paired reads	8,112,913	8,260,474
Total length (bp)	1,216,936,950	1,239,071,100
Predicted genes (loci)	15,939	19,501
Known	10,087	10,469
Novel	5,852	9,032
Predicted transcripts	21,009	25,706
Known	14,301	18,931
Novel	6,708	6,775
Multi-transcript loci	3,905	4,677
Transcripts per locus	~1.32	~1.32

Summary statistics of the reads and the reads’ total length from the two libraries female (Sjc-F) or male (Sjc-M) parasite were listed.

### Alternative splicing in *S. japonicum*

Four types of alternative splicing in both female and male worms of *S. japonicum* (Table 2 and Additional file 5) were identified, including ES, IR, ADS, and AAS. Of the alternative splicing events, AAS and ADS were more common than the other two types, suggesting that the gene regulation mechanism of the *Schistosoma* parasite is diverged from that of the mammalian taxa, in which ES has been more commonly observed [19]. In female *S. japonicum*, a total of 13,438 alternative splicing events were bioinformatically predicted while in male worms, a total of 16,507 were predicted (Table 2). The percentage of different alternative splicing events was similar between the two sexes (Table 2); however, the genes undergoing alternative splicing were not necessarily the same between them (Additional file 5).

**Table 2:** **Statistics for alternative splicing events**

Alternative splicing class	Sj-F vs reference*		Sj-M vs reference*		Sj-F vs Sj-M
		Percentage (%)		Percentage (%)	
# of loci with alternative splicing	5,574		6,442		6,453
Total alternative splicing events	13,438	100.0	16,507	100.0	18,734
Alternative donor site	2,933	21.8	3,578	21.7	4,252
Alternative acceptor site	3,096	23.0	3,800	23.0	5,135
Intron retained	2,814	20.9	3,270	19.8	3,960
Exon skip	2,594	19.3	3,296	20.0	3,961
Exon new	2,001	14.9	2,563	15.5	1,426
5′ Extend	5,263	39.2	6,221	37.7	4,872
3′ Extend	4,322	32.2	4,966	30.1	4,085

Sj-F and Sj-M are the detected alternative splicing events of the female (Sjc-F) or male (Sjc-M) parasite.

Reference* is the reference annotated genes of the two libraries for the female (Sjc-F) or male (Sjc-M) parasite.

**Figure 3:**
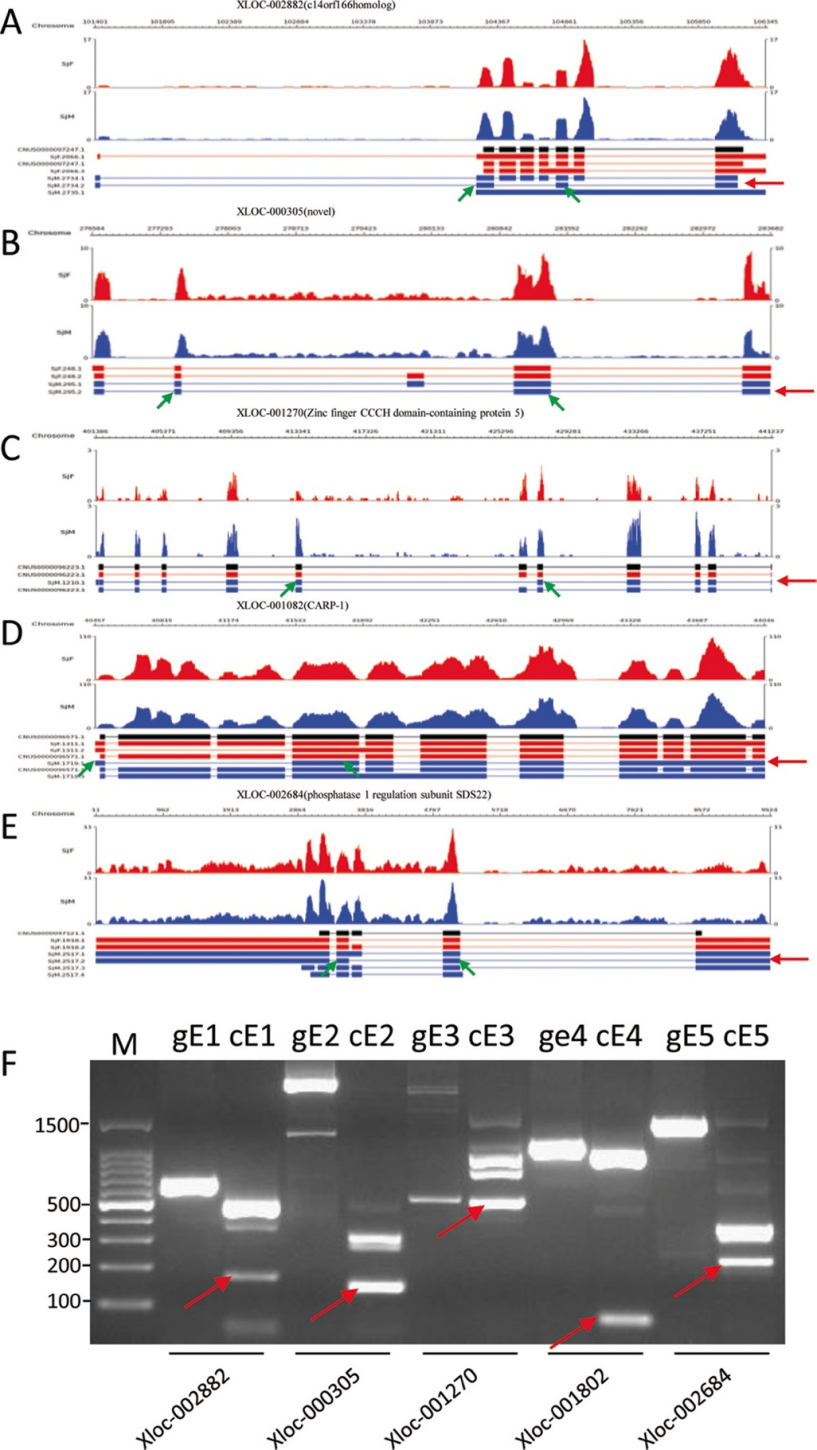
**Sequence mapping and verification of 5 genes with exon skipping events detected by RNA-seq by PCR and RT-PCR.** The expression profiles of the same gene in female (red) and male (blue) parasites were placed under the line representing the chromosome position. The black lines represent original annotated gene structures (thick lines indicate exonic regions, and thin lines indicate intronic regions), while the active transcripts in red and blue identified from the same genes in female and male parasites are underneath. The five genes and transcripts **(A, B, C, D, E)** with exon skipping events were confirmed by PCR and RT-PCR **(F)**. gE indicates PCR products amplified from genomic DNA, and cE indicates PCR products amplified from cDNA. Red arrows indicate transcripts generated by exon skipping, and green arrows indicate primer locations.

**Figure 4:**
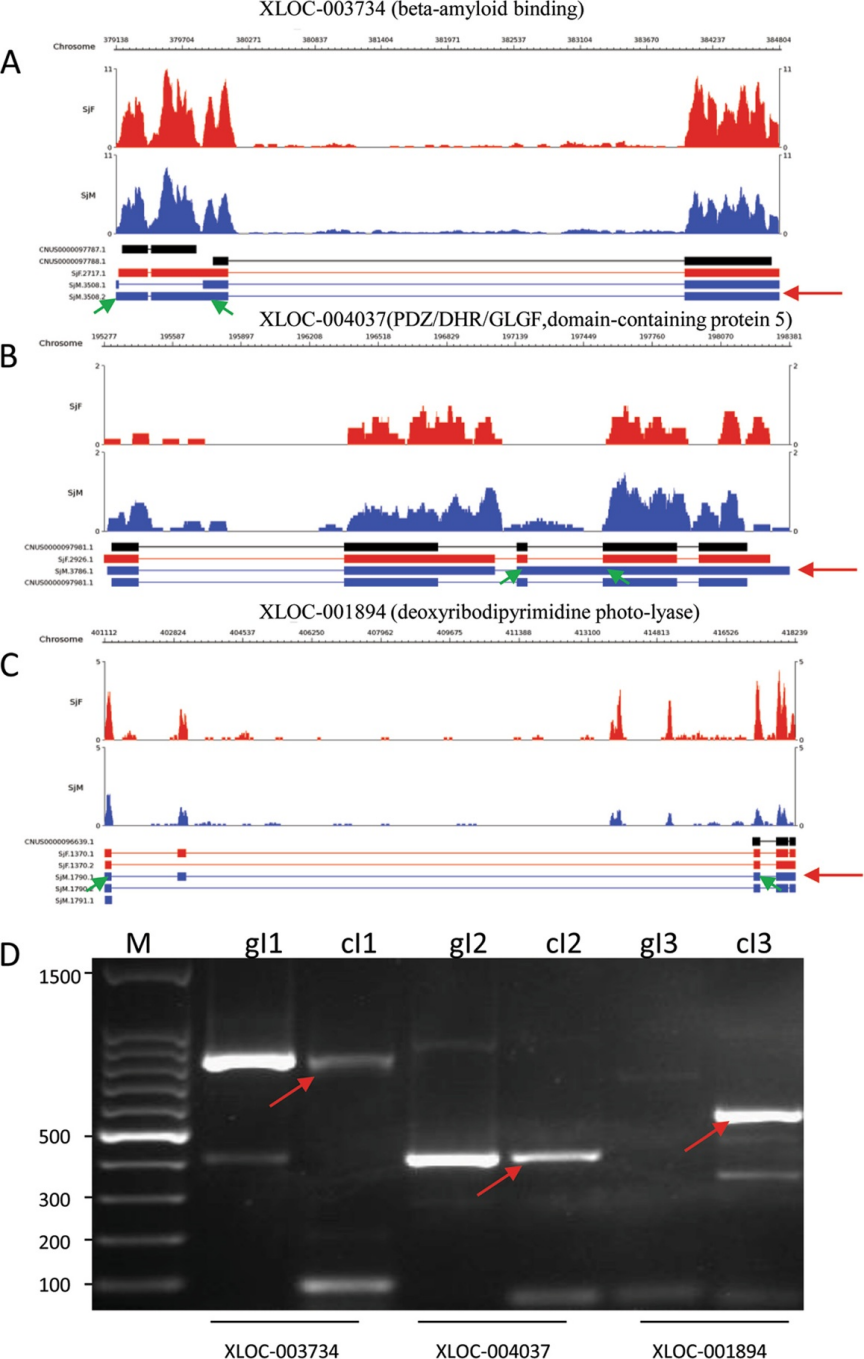
**Sequence mapping and verification of three genes with intron retention events detected by RNA-seq by PCR and RT-PCR.** The three genes and transcripts **(A, B, C)** with intron retention events were confirmed by PCR and RT-PCR **(D)**. gI indicates PCR products amplified from genomic DNA, and cI indicates PCR products amplified from cDNA. Red arrows indicate transcripts generated by intron retention, and green arrows indicate primer locations.

To confirm experimentally the prediction of the alternative splicing events in the bioinformatic analysis, eight transcripts in which alternatively spliced fragments were more than 100 bp were chosen randomly. Transcripts generated by ES skipping of five genes and transcripts generated by IR of three genes were validated by PCR and RT-PCR. The five genes with ES activity included one that encodes the protein C14orf166 homolog; a novel gene; *S. japonicum* Zinc finger CCCH domain-containing protein 5; *S. japonicum* cell division cycle and apoptosis regulator protein 1; and *S. japonicum* protein phosphatase 1 regulatory subunit SDS22. The three genes with IR were respectively beta-amyloid binding protein (Sjc_0025470), *S. japonicum* IPR001478 PDZ/DHR/GLGF domain-containing protein, and deoxyribodipyrimidine photolyase. The amplicons of all RT-PCR reactions were cloned and sequenced and were correlated with the predicated alternative splicing events (Figures 3 and 4), suggesting that the bioinformatic prediction based on the primary sequencing data was reliable.

### Functional category of alternatively spliced genes in *S. japonicum*

After mapping of the RNA-Seq reads to the *S. japonicum* reference genome, transcripts were assembled and their relative abundances were calculated. Cuffdiff was used to find significant changes in gene level expression in the two libraries [27]. Genes subject to alternative splicing and showing significant differences in expression in the two libraries are listed in Table 3 and Additional file 6: Figure S2. Genes related to the function of genetic information processing were found to be more biased to the female parasite while genes related to the environmental information processing were more active in the male parasites (Additional file 7). These data reflect the biology of the two sexes of the parasite. The female parasites, which are kept in the cavity of the male parasites, are more active in the reproduction process while the male parasites are principally responsible in the host–parasite interaction.

**Table 3:** **GO classification statistics of alternatively processed genes that were differentially expressed in female and male** ***S. japonicum***

Function		Total AS & Diff	P value
Cellular component	Cell part	217	0.501094
	Envelope	1	0.145179
	Extracellular region	15	0.000048
	Macromolecular complex	43	0.670975
	Membrane\-enclosed lumen	0	0.069725
	Organelle	78	0.89208
	Synapse	2	0.906739
Molecular function	Antioxidant activity	0	0.314056
	Auxiliary transport protein activity	0	0.693184
	Binding	262	0.44144
	Catalytic activity	161	0.017811
	Electron carrier activity	2	0.987565
	Enzyme regulator activity	20	0.100961
	Metallochaperone activity	0	0.780276
	Molecular transducer activity	12	0.900991
	Obsolete molecular function	7	0.030106
	Proteasome regulator activity	0	0.780276
	Structural molecule activity	30	0.000203
	Transcription regulator activity	21	0.609164
	Translation regulator activity	0	0.119509
	Transporter activity	38	0.863425
Biological process	Anatomical structure formation	5	0.221865
	Biological adhesion	24	0
	Biological regulation	69	0.706126
	Cell killing	0	0.780276
	Cellular component biogenesis	5	0.110686
	Cellular component organization	7	0.066938
	Cellular process	208	0.019832
	Death	0	0.235977
	Developmental process	4	0.952945
	Establishment of localization	52	0.723642
	Growth	0	0.780276
	Immune system process	1	0.563582
	Localization	53	0.716675
	Locomotion	1	0.370986
	Metabolic process	175	0.004955
	Multi\-organism process	0	0.576811
	Multicellular organismal process	4	0.624764
	Obsolete biological process	2	0.002313
	Pigmentation	69	0.967452
	Reproduction	0	0.576811
	Response to stimulus	10	0.673491

*AS* alternative splicing. *Diff* differentially expressed.

Total AS & Diff, genes with both alternative splicing and differential expression in female and male *S. japonicum.*

### Identification of novel transcripts from intergenic regions and previously determined pseudogenes

**Figure 5:**
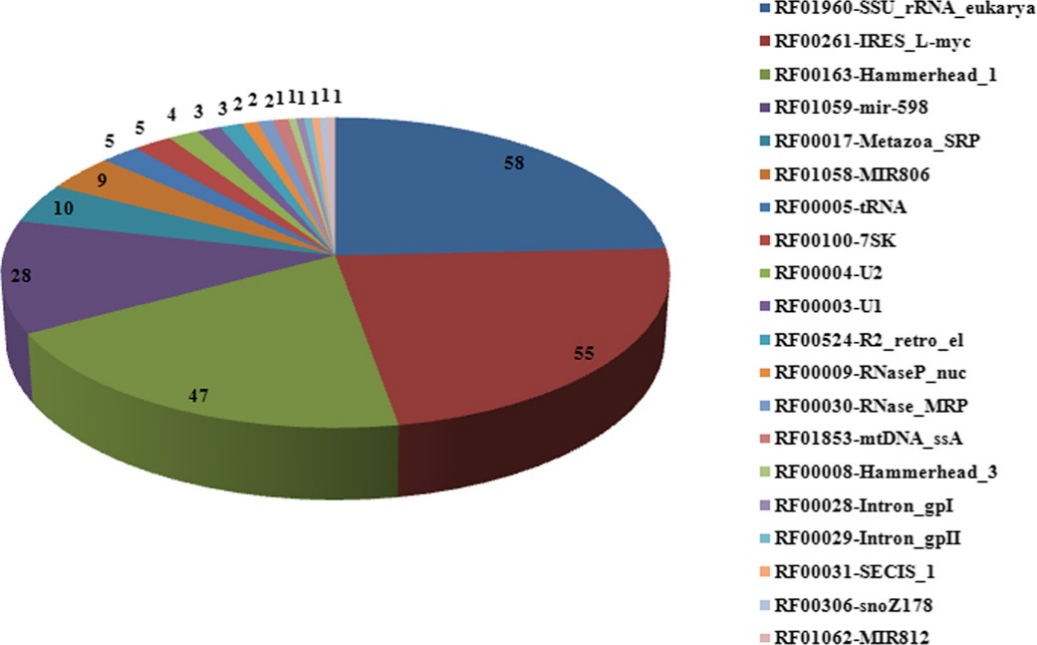
**Numbers of non-coding RNA transcripts identified in the transcriptomes of female and male** ***S. japonicum*** **.**

One of the advantages of transcriptomic analysis is that it allows identification of novel transcripts that may not be predicted based on genomic sequences. The novel sequences can thus provide a powerful tool for re-annotation of the genome of *S. japonicum*, which has been poorly assembled (7). Of the novel polyadenylated sequences, two classes of transcripts have been identified: one that does not map either to regions of the genome corresponding to annotated genes or to the untranslated regions, and another that maps to previously annotated pseudogenes [7]. We identified 9,286 novel transcripts that completely matched the previously annotated intergenic regions of the genome. The length of these transcripts was from 74 to 166,115 bp, with an average length of 1,965 bp (Additional file 8). It has been reported that the *S. mansoni* genome contains many small open reading frames (8). Our results indicated that the small transcripts derived from both intergenic and "pseudogenes" in *S. japonicum* may encode important functions, as reported for the human genome [38]. Further, 31% (2,851 sequences) of these transcripts had at least one complete open reading frame that could be translated into proteins; the other 69% were not annotated (Additional file 8). Thus, of the 9,286 novel transcripts, at least 2,851 genomic sequences corresponding to the transcripts can be re-annotated as protein-coding genes. A total of 239 transcripts were mapped to the non-coding RNA database of the Rfam [39–43], which is also frequently used as a source of high-quality alignments for training and benchmarking RNA sequence analysis software tools. These transcripts were found as either microRNAs or ribosomal or other non-coding RNAs (Figure 5, Additional files 9 and 10). They were likely the contaminated sequences which were not completely depleted during mRNA purification process.

**Figure 6:**
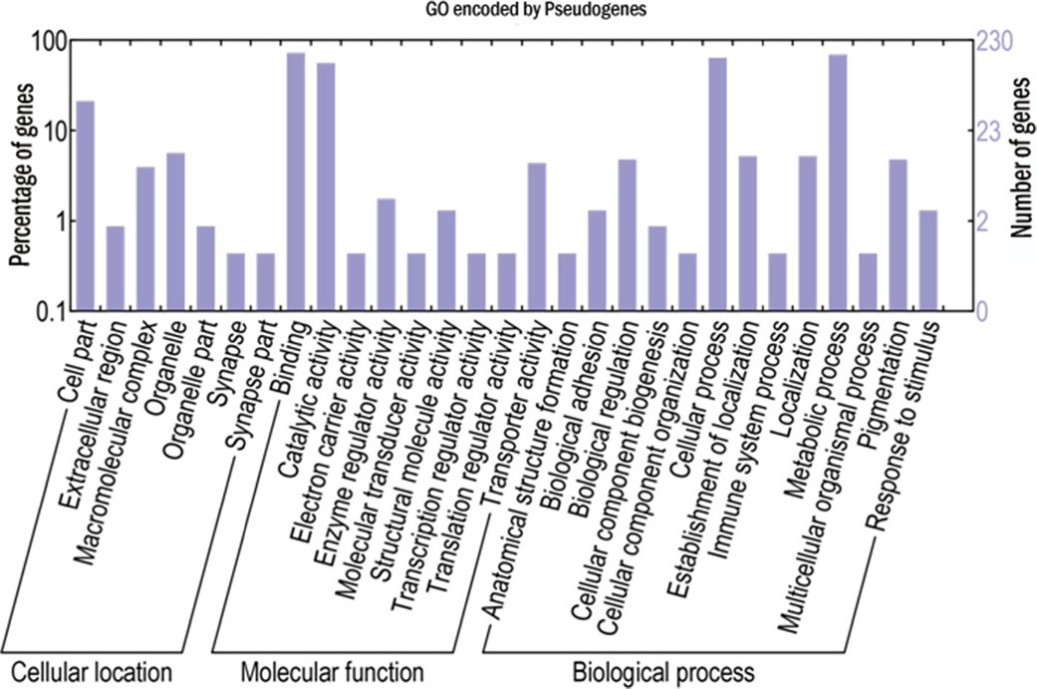
**GO categories of transcripts with a complete open reading frame and derived from pseudogenes.** The percentages of the transcripts encoding the same proteins with similar function are indicated on the left while the numbers of the transcripts identified are indicated on the right.

Furthermore, among the 9,286 novel transcripts, we detected 1,392 that were derived from pseudogenes; of these, 690 were derived from annotated pseudogenes, and the rest were from unannotated pseudogenes (Additional files 9 and 10). Pseudogenes can be transcribed from either direction, which contributed to the templates for generation of small endogenous interfering RNAs in *S. japonicum*, which is more common in transposable elements [21–23]. The identification of these pseudogene-derived transcripts suggested that all sequences are polyadenylated and that there is no discrimination between coding and non-coding transcripts in the RNA polyadenylation process in *Schistosoma* parasites. On the other hand, complete reading frames were indeed identified in a number of pseudogene-derived transcripts that encoded proteins with known functions (Figure 6). Thus, these "pseudogene" genes can be re-annotated as protein-coding genes.

## Conclusions

In summary, by using RNA-seq technology, we obtained the global transcriptomes of male and female *S. japonicum*. Approximately 80% of the total reference genes ( http://lifecenter.sgst.cn/schistosoma/en/schdownload.do) were expressed in the adult stage of the parasite, representing the majority of the transcriptomes. These results further provide a comprehensive view of the global transcriptome of *S. japonicum.* The findings of a substantial level of alternative splicing events dynamically occurring in the parasitization in the mammalian hosts of the *S. japonicum* suggest complicated transcriptional and post-transcriptional regulatory mechanisms employed by the parasite. The data should not only significantly improve the re-annotation of the genome sequences but also should provide new information about the biology of the parasite.

## Electronic supplementary material

Additional file 1: **Differentially expressed genes between females and males.** (XLS 605 KB)

Additional file 2: Figure S1: Junction sites. (TIFF 826 KB)

Additional file 3: Table S1: Primers and sequences for verification of the alternative splicing events. (DOC 36 KB)

Additional file 4: **Total transcripts identified in female and male parsites.** (XLS 9 MB)

Additional file 5: **Genes with alternative splicing in female and male parsites.** (XLS 700 KB)

Additional file 6: Figure S2: Go category of the genes that were alternatively spliced and also differentially transcribed. (TIFF 144 KB)

Additional file 7: **Genes related to the function of genetic information processing and environmental information.** (XLSX 38 KB)

Additional file 8: **Novel transcripts identified.** (XLS 6 MB)

Additional file 9: **Noncoding transcripts identified in the female** ***S. japonicum*** **.** (XLS 62 KB)

Additional file 10: **Noncoding transcripts identified in the male** ***S. japonicum.*** (XLS 64 KB)
