# Supplementary figures and images for: Genome-wide transcriptome analysis shows extensive alternative RNA splicing in the zoonotic parasite Schistosoma japonicum
Source: BMC Genomics. 2014 Aug 26;15(1):715. doi: 10.1186/1471-2164-15-715 (PMC4203478; doi:10.1186/1471-2164-15-715)

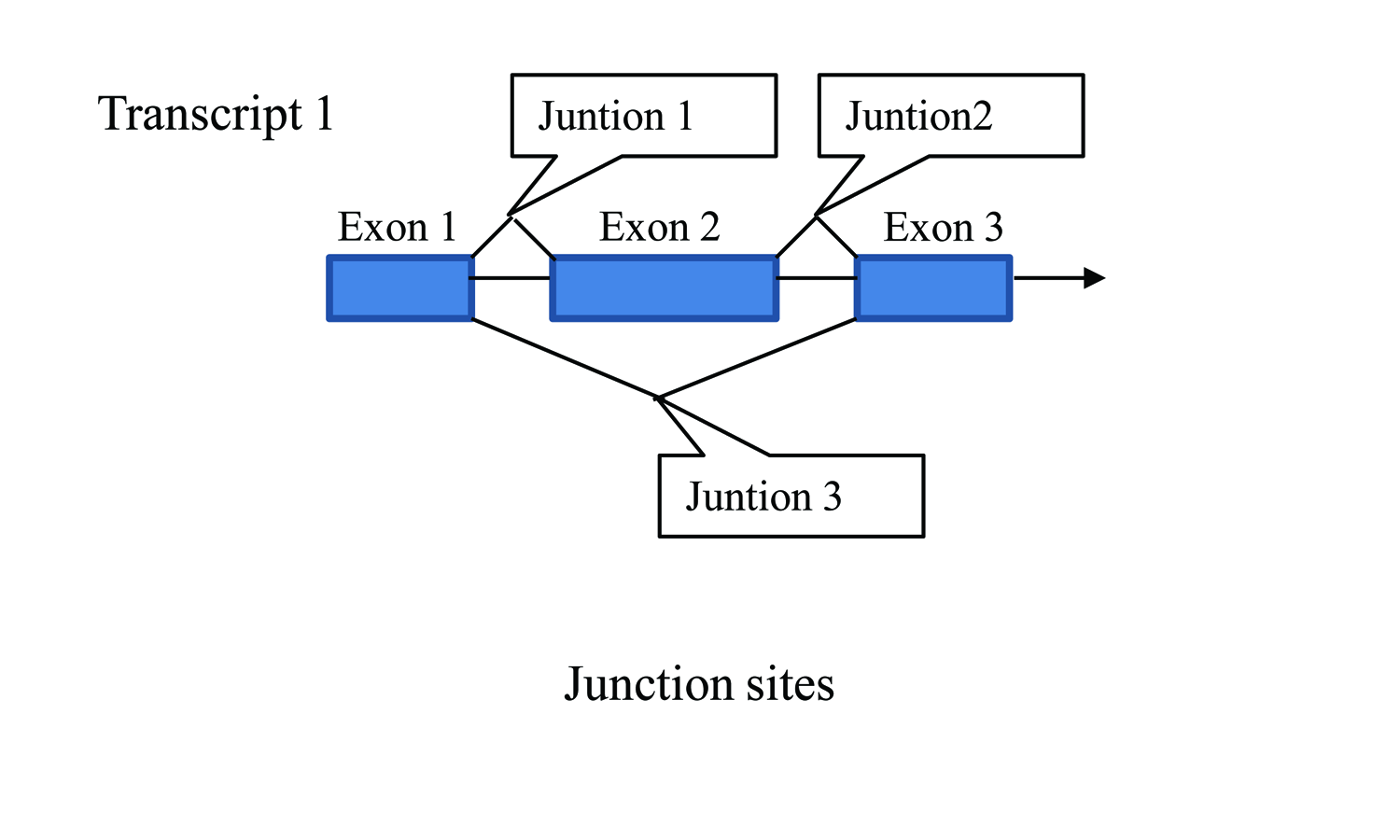

Supplement: Supplementary file 2 — Additional file 2: Figure S1: Junction sites. (TIFF 826 KB) [file 12864_2014_6414_MOESM2_ESM.tiff]

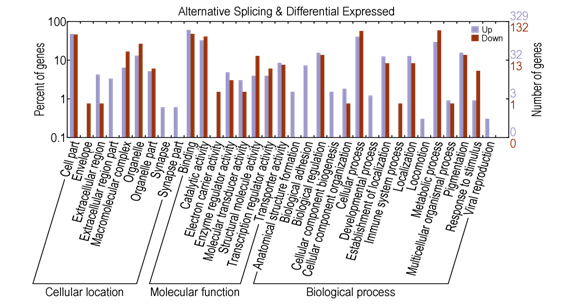

Supplement: Supplementary file 6 — Additional file 6: Figure S2: Go category of the genes that were alternatively spliced and also differentially transcribed. (TIFF 144 KB) [file 12864_2014_6414_MOESM6_ESM.tiff]
