# Supplementary material for: Genome-wide transcriptome analysis shows extensive alternative RNA splicing in the zoonotic parasite Schistosoma japonicum
Source: BMC Genomics. 2014 Aug 26;15(1):715. doi: 10.1186/1471-2164-15-715 (PMC4203478; doi:10.1186/1471-2164-15-715)
Supplement: Supplementary file 3 — Additional file 3: Table S1: Primers and sequences for verification of the alternative splicing events. (DOC 36 KB) [file 12864_2014_6414_MOESM3_ESM.doc]

**Additional file 3: Table S1. PCR primer sequences.**

| name | primer | sequence |
| --- | --- | --- |
| gE1, cE1 | Forward primer | CACAACTTCCGCATTAGA |
|  | Reverse primer | TGGGTGGTTAGGTATTTGTAGT |
| gE2, cE2 | Forward primer | ATAACCGCATCCTCCCTA |
|  | Reverse primer | CACCTCGGTAGAAGCCAC |
| gE3, cE3 | Forward primer | AAATCAATCAAGTGGTCGTG |
|  | Reverse primer | GACAGTGCCAACAGAAGG |
| gE4, cE4 | Forward primer | GCGAATCGTTGGGGTCGTAA |
|  | Reverse primer | GCTTTGGTGTCAGGCATA |
| gE5, cE5 | Forward primer | TACAAAACAATCGTATAAGTCG |
|  | Reverse primer | TATGGAACTCTTTAAGGTTTGAC |
| gI1, cI1 | Forward primer | CTTGGTCTTAATAATTATGC |
|  | Reverse primer | GGGTGTCGTCGTCAAGC |
| gI2, cI2 | Forward primer | AAACCTTACAGTGCGAAAA |
|  | Reverse primer | AGAACTGCGAAGACCCA |
| gI3, cI3 | Forward primer | TCCCACCTCTTATTAAACACCCCTG |
|  | Reverse primer | CTGTCCGCACCAGTTGGCGT |
